# Synthesis and Evaluation of Baylis-Hillman Reaction Derived Imidazole and Triazole Cinnamates as Antifungal Agents

**DOI:** 10.1155/2018/5758076

**Published:** 2018-10-16

**Authors:** Grady L. Nelson, Michael J. Williams, Shirisha Jonnalagadda, Mohammad A. Alam, Gautam Mereddy, Joseph L. Johnson, Sravan K. Jonnalagadda

**Affiliations:** ^1^Integrated Biosciences Graduate Program, University of Minnesota, Duluth, MN 55805, USA; ^2^Department of Chemistry and Biochemistry, University of Minnesota, Duluth, MN 55805, USA

## Abstract

Allylic acetates derived from Baylis-Hillman reaction undergo efficient nucleophilic isomerization with imidazoles and triazoles to provide imidazolylmethyl and triazolylmethyl cinnamates stereoselectively. Antifungal evaluation of these derivatives against* Cryptococcus neoformans* exhibits good minimum inhibitory concentration values. These compounds exhibit low toxicity in proliferating MCF-7 breast cancer cell line. Structure activity relationship studies indicate that halogenated aromatic derivatives provide better antifungal activity.

## 1. Introduction

**Figure 1:**
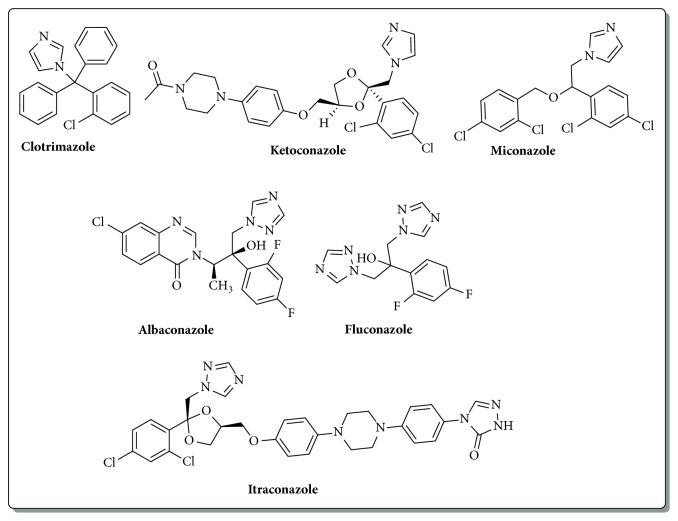
Few examples of imidazole and triazole based antifungal agents.

Due to the advances in modern medicine, overall life expectancy is being greatly extended; however the unintended consequence is a large increase in reduced immune system and immunocompromised cancer and organ transplant patients. This extension of life expectancy resulted in a lot of opportunistic fungal and bacterial infections often leading to patient mortality. Imidazole and triazole based antifungal agents have been the mainstay of the treatments for fungal infections (Figure 1) [1–4]. With the advent of resistance to many of the clinically used drugs, novel candidate compounds that can overcome the resistance are urgently required. In this regard, we envisioned to develop novel imidazole and triazole based small molecules that could be easily synthesized and densely functionalized for structure activity relationship studies.

*Cryptococcus neoformans* consists of three variants:* C. neoformans* var.* grubii*,* C. neoformans* var.* neoformans*, and* C. neoformans* var.* gattii*. The variants* grubii* and* gattii* are mainly responsible for most cases of pathogenic infections in both immunocompromised and sometimes even immunocompetent patients [5–11]. Without a proper treatment, this infection will invariantly lead to patient morbidity and mortality. The standard treatment includes amphotericin-B infusion in combination with 5-flucytosine to reduce the fungal infection followed by maintenance therapy with azoles such as fluconazole. Literature reports indicate that* Cryptococcus neoformans* is sensitive to fluconazole [5–11]. In many developing and poor countries, the usage of amphotericin-B and 5-flucytosine adds a lot of cost burden and treatment may be mainly limited to high-dose azoles. These drugs have many side effects such as hepatotoxicity and myelosuppression [9]. However, resistance to azole based therapy has been detected in some* Cryptococcus *based infections [8] and hence development of novel and inexpensive azole based therapeutics will be highly beneficial for the treatment of* Cryptococcus *and other fungal based infections.

**Figure 2:**
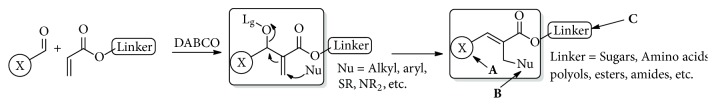
Baylis-Hillman reaction template.

The Baylis-Hillman (BH) reaction is an important carbon-carbon bond forming synthetic transformation that provides highly substituted allylic alcohols and amines in one step [12–16]. Nucleophilic substitution on the BH derived allylic alcohols can be carried out through the conversion of the alcohol unit to the bromide or acetate leaving groups (Figure 2). BH reaction template also offers high tunability at three different places. This reaction has been extensively investigated for various types of pharmaceutical development [17–24].

The isomerization of BH acetates or bromides with imidazole or triazoles provides facile access to functionalized allyl imidazoles or allyl triazoles [25–29]. This protocol allows high flexibility and rapid access to structurally diverse functionalized allyl imidazoles and triazoles. If these molecules exhibit any antifungal activity, a new class of imidazole and triazole based antifungal agents will be constituted. With these goals in mind, we undertook a project on the development of novel functionalized imidazoles and triazoles utilizing BH chemistry and our results are reported below.

## 2. Materials and Methods

### 2.1. Representative Procedure for Isomerization of Baylis-Hillman (BH) Acetates

#### 2.1.1. Synthesis of Methyl (*E*)-2-((1H-imidazol-1-yl)methyl)-3-(4-chlorophenyl)acrylate (**12**)

To a stirred solution of BH acetate (5 mmol) in 1:1 THF-water (10 mL) was added imidazole (7.5 mmol) at room temperature and stirred overnight. The reaction mixture was extracted three times with ethyl acetate-water and the combined organic layers were dried using MgSO_4_. The dried organic layer was concentrated in vacuum and purified via silica gel column chromatography to obtain the pure methyl (*E*)-2-((1H-imidazol-1-yl)methyl)-3-(4-chlorophenyl)acrylate** 12** in 63% yield. ^1^H NMR (500MHz, CDCl_3_) *δ* 7.96 (s, 1 H), 7.45 (s, 1 H), 7.39 (d,* J* = 8 Hz, 2 H), 7.24 (d,* J* = 8 Hz, 2 H), 7.01 (s, 1 H), 6.84 (s, 1 H), 4.94 (s, 2 H), 3.80 (s, 3 H) ppm; ^13^C NMR (125 MHz, CDCl_3_) *δ* 166.9, 143.7, 137.2, 136.1, 132.5, 130.4, 129.6, 127.8, 118.9, 52.8, 43.1 ppm. Anal. Calcd for C_14_H_13_ClN_2_O_2_ (276.72): C 60.77, H 4.74, N 10.12 Found: C 60.47, H 4.71, N 9.93.

#### 2.1.2. Synthesis of Methyl (*E*)-2-((1H-1,2,4-triazol-1-yl)methyl)-3-(4-fluorophenyl)acrylate (**24**)

The same procedure was followed and imidazole was replaced with 1,2,4-triazole. Compound** 24** yield: 55%; ^1^H NMR (500 MHz, CDCl_3_) *δ* 8.28 (s, 1 H), 8.03 (s, 1 H), 7.98 (s, 1 H), 7.80-7.78 (m, 2 H), 7.17 (t,* J *= 8.25 Hz, 2 H), 5.19 (s, 2 H), 3.83 (s, 3 H) ppm; ^13^C NMR (125 MHz, CDCl3) *δ* 167.1, 164.8, 162.8, 152.1, 144.5, 132.1, 130.1, 125.2, 116.4, 116.3, 52.8, 46.2 ppm; Anal. Calcd for C_13_H_12_FN_3_O_2_ (261.26): C 59.77, H 4.63, N 16.08 Found: C 59.96, H 4.69, N 16.00.

### 2.2. Disk Diffusion Susceptibility Testing

Kirby-Bauer testing was used as the initial screen for determining the antifungal activity of the compounds.* Cryptococcus neoformans* was obtained from the ATCC (ATCC 32045). Isolates were cultured in phenol red free RPMI medium supplemented with 3% glucose (modified as indicated in the CLSI protocol M27-A3). The culture was grown up to an OD (0.4-0.5) at 530 nm giving an inoculum stock of 1 x 10^6^ to 5 x 10^6^ cells per mL. Sterile swabs were used to spread the inoculum evenly on LB agar plates containing ampicillin. 5 *μ*L of each 100 mM DMSO stock solution was added to sterile 1 cm diameter Whatman filter discs and then placed into a quadrant of a freshly streaked plate. The zone of inhibition was measured after 24 and 48 hours. Each compound was tested in at least triplicate, and fluconazole was used as a positive control.

### 2.3. Minimum Inhibitory Concentration (MIC) Assay

MIC assays were performed on samples that showed zones of inhibition greater than 1.5 cm. A fresh stock inoculum was diluted 1000-fold in modified RPMI medium. Each 28 mg/mL mM DMSO stock solution was diluted to 56 *μ*g/mL. 100 *μ*L of the diluted* C. neoformans* inoculum was added to each well of a round-bottom 96-well plate containing twofold serial dilutions of each stock antifungal compound solution beginning at 28 *μ*g/mL with a DMSO-only control in the final well of each row. The plates were incubated at 37°C in a non-CO_2_ incubator, and the absorbance at 600 nm was measured using a plate reader after 24 and 48 hours. The reported MICs corresponded to the lowest compound dilution that significantly inhibited growth (more than 50% relative to control). Each compound was tested in at least triplicate, and fluconazole and miconazole were used as positive controls, returning MIC values consistent with published data.

### 2.4. Sulforhodamine-B Cytotoxicity Assay

A standard sulforhodamine-B assay [30, 31] was used to evaluate the cytotoxicity of the synthesized compounds on proliferating cancer cells. MCF-7 cells were cultured in 5% CO_2_ atmosphere at 37°C in Iscove's Modified Dulbecco's medium containing 10% FBS and 1% antibiotic (penicillin-streptomycin). MCF-7 cells were seeded at a concentration of approximately 5x10^5^ cells/mL in 48 well plates such that each well contains 400*μ*L of media and incubated for 24 hours. The test compounds were initially diluted in DMSO and diluted 1000 times in growth media so that the final DMSO concentration was <0.1%. Growth media were removed from 48 well plates and test compounds in 400*μ*L of growth media were added to the wells. Paclitaxel, DMSO, and growth media were used as controls. The plates were incubated for 72 hours. After removing the media, the cells were washed with 1% Dulbecco's phosphate-buffered saline and dried. 100*μ*L of 0.5% sulforhodamine-B (SRB) in 1% acetic acid was added in each well and incubated at 37°C for 45 minutes. SRB solution was removed and the wells were washed 5 times with 1% acetic acid solution and dried. The cells were dissolved in 400*μ*L of 10mM Tris base (pH 10.2) and absorbance was recorded at 540nm. The absorbance is directly proportional to the cellular protein.

## 3. Results and Discussion

**Scheme 1:**
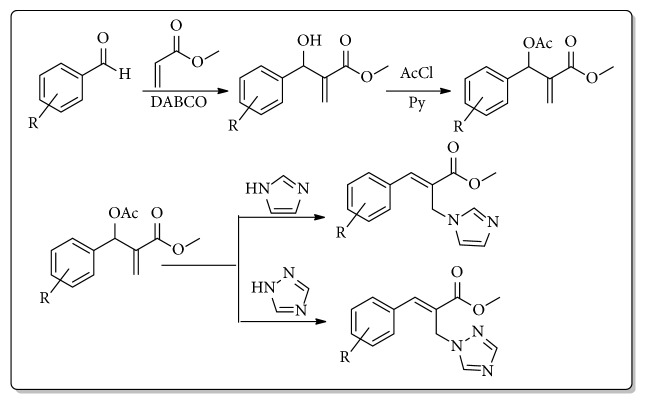
Synthesis of 2-(imidazolylmethyl) and 2-(triazolylmethyl) cinnamates.

We initiated the synthesis of BH derived allyl alcohols with various aromatic aldehydes with methyl acrylate in the presence of DABCO. The reactions took place smoothly in all these cases and the product alcohols were obtained in good yields upon silica gel column chromatography (70-85%). These alcohols were converted into acetates by treatment with acetyl chloride in the presence of triethylamine or pyridine. Reaction of these acetates with imidazole/triazole in THF and water at room temperature led to the formation of corresponding cinnamoyl imidazoles/triazoles** 1-26** (Scheme 1, Table 1). The compounds were characterized by proton and carbon NMR spectroscopy (Supplementary Materials (available here)). In all the cases, the* E*-isomer of the imidazole/triazole olefins was predominantly obtained (>95%) based on crude NMR analysis.

## 4. Biological Evaluation

After synthesizing BH derived functionalized imidazoles and triazoles, we then evaluated the antifungal efficacy of all these derivatives on a representative fungal species* Cryptococcus neoformans*. Initial antifungal activities were carried out using Kirby-Bauer disk method. This method is rapid and provides qualitative information about the biological activity of the molecules. Gratifyingly, many of the derivatives were found to be active with good to moderate zone of inhibition values (1-3.7 cm). The halo of 1 cm means that there was no inhibition. The top five compounds (**2, 11, 12, 13, and 24**) that exhibited highest zone of inhibition were further evaluated for minimum inhibitory concentration (MIC) values. The most active compounds were tested in at least triplicate using a 96-well plate in serial dilutions. Fluconazole and miconazole were used as positive controls, returning MIC values consistent with published data. Some of the tested compounds exhibited good activity against* Cryptococcus neoformans* (~8-13 *μ*g/mL, Table 2).

We then evaluated for the cytotoxicity of these compounds using a breast cancer cell line MCF-7. Sulforhodamine-B assay was used to determine the cytotoxicity profile of these compounds. All the compounds were tested in triplicate in 48-well plates. None of the compounds were toxic below 50 *μ*M concentration in proliferating cells indicating the utility of these imidazole and triazole derivatives for further development.

## 5. Conclusions

In conclusion, we synthesized several imidazoles and triazoles containing small molecules derived from BH reaction template. The synthesized molecules including the starting materials were evaluated for their antifungal activity against* C. neoformans*. Several halogenated aromatic imidazole and triazole derivatives exhibited promising antifungal activity against* C. neoformans*. It is also encouraging to note that the active molecules exhibited little to no cytotoxicity when tested on proliferating breast cancer cell line MCF-7. The compounds are easy to synthesize and exhibit selective antifungal activity against* C. neoformans*. We believe that this work should attract the attention of medical and pharmaceutical scientists for further structure activity investigation and development of more potent analogs for clinical use.

## Figures and Tables

**Table 1:** 2-(imidazolylmethyl) and 2-(triazolylmethyl) cinnamates.

Compound Number	Compound	% yield
**1**	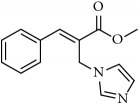	55
**2**	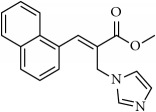	86
**3**	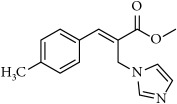	76
**4**	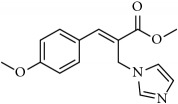	83
**5**	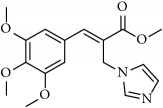	89
**6**	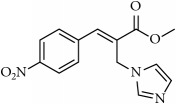	78
**7**	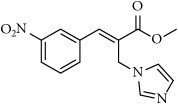	82
**8**	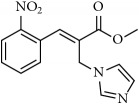	75
**9**	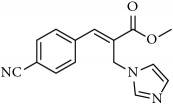	70
**10**	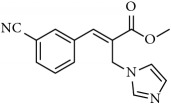	71
**11**	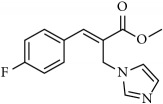	60
**12**	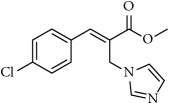	63
**13**	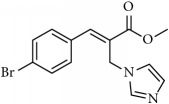	70
**14**	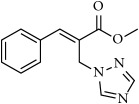	68
**15**	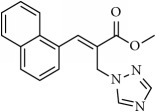	84
**16**	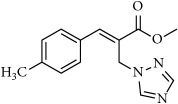	75
**17**	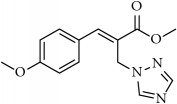	82
**18**	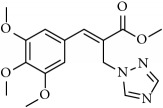	88
**19**	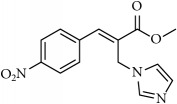	80
**20**	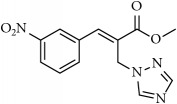	75
**21**	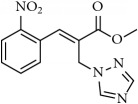	76
**22**	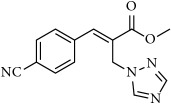	57
**23**	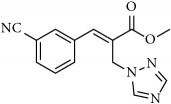	57
**24**	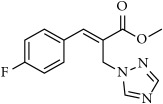	55
**25**	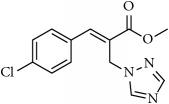	78
**26**	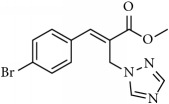	78

**Table 2:** Minimum inhibitory concentrations (MIC50*∗*) of lead molecules in *μ*g/mL.

Compound Number	Compound	*Cryptococcus neoformans*
2	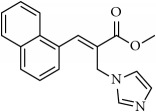	8±4

**11**	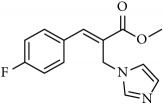	11±4

**12**	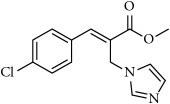	9±4

**13**	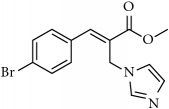	9±4

**24**	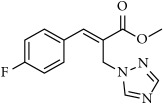	13±5

	Fluconazole	3±1

	Miconazole	< 0.5

*∗*Average±SEM of three separate experiments.

## Data Availability

An experimental procedure describing the synthesis of compounds, along with data with critical compounds, has been provided in the manuscript for others to utilize for their research.

## Supplementary Materials

Supplementary Materials NMR spectroscopic data and elemental analysis of all the synthesized compounds are included in the Supplementary Materials.
