# Supplementary material for: Synthesis and Evaluation of Baylis-Hillman Reaction Derived Imidazole and Triazole Cinnamates as Antifungal Agents
Source: Int J Med Chem. 2018 Oct 16;2018:5758076. doi: 10.1155/2018/5758076 (PMC6206569; doi:10.1155/2018/5758076)
Supplement: Supplementary Materials — NMR spectroscopic data and elemental analysis of all the synthesized compounds are included in the Supplementary Materials. [file 5758076.f1.docx]

*Spectral Characterization*

*Methyl (E)-2-((1H-imidazol-1-yl)methyl)-3-phenylacrylate* **1**

**^1^H NMR (500MHz, CDCl_3_):** δ 8.06 (s, 1H), 7.50 (s, 1H), 7.48-7.38 (m, 3H), 7.37-7.32 (m, 2H), 7.05 (s, 1H), 6.88 (s, 1H), 5.00 (s, 2H), 3.83 (s, 3H)

**^13^C NMR (125MHz, CDCl_3_):** δ 167.2, 145.1, 137.3, 134.2, 129.9, 129.4, 129.3, 129.1, 127.3, 119.0, 52.8, 43.2

**Anal. Calcd for C_14_H_14_N_2_O_2_** (242.28): C, 69.41; H, 5.82; N, 11.56; **Found:** C, 69.74; H, 5.81; N, 11.59

*Methyl (E)-2-((1H-imidazol-1-yl)methyl)-3-(naphthalen-1-yl)acrylate* **2**

**^1^H NMR (500MHz, CDCl_3_):** δ 8.49 (s, 1H), 7.94 (m, 2H), 7.82-7.88 (m, 1H), 7.57-7.61 (m, 2H), 7.49-7.55 (m, 1H), 7.36 (s, 1H), 7.28 - 7.32 (m, 1H), 6.96 (s, 1H), 6.73 (s, 1H), 4.87 (s, 2H), 3.89 (s, 3H)

**^13^C NMR (125MHz, CDCl_3_):** δ 166.8, 143.7, 137.6, 133.8, 131.6, 131.3, 130.2, 130.0, 129.4, 129.0, 127.4, 127.0, 126.3, 125.5, 124.6, 119.2, 52.8, 43.3

**Anal. Calcd for C_18_H_16_N_2_O_2_** (292.34): C, 73.95; H, 5.52; N, 9.58; **Found:** C, 73.68; H, 5.47; N, 9.64

*Methyl (E)-2-((1H-imidazol-1-yl)methyl)-3-(p-tolyl)acrylate* **3**

**^1^H NMR (500MHz, CDCl_3_):** δ 8.04 (s, 1H), 7.51 (s, 1H), 7.26 (m, 4H), 7.05 (s, 1H), 6.91 (s, 1H), 5.02 (s, 2H), 3.39 (s, 3H), 2.42 (s, 3H)

**^13^C NMR (125MHz, CDCl_3_):** δ 167.4, 145.3, 140.5, 137.3, 131.3, 130.0, 129.6, 129.3, 126.3, 119.0, 52.7, 43.3, 21.7

**Anal. Calcd for C_15_H_16_N_2_O_2_** (256.31): C, 70.29; H, 6.29; N, 10.93; **Found:** C, 70.46; H, 6.31; N, 10.89

*Methyl (E)-2-((1H-imidazol-1-yl)methyl)-3-(4-methoxyphenyl)acrylate* **4**

**^1^H NMR (500MHz, CDCl_3_):** δ 8.02 (s, 1H), 7.54 (s, 1H), 7.33 (d, *J* = 10.0 Hz, 2H), 7.07 (s, 1H), 6.97-6.94 (m, 3H), 5.04 (s, 2H), 3.87 (s, 3H), 3.83 (s, 3H)

**^13^C NMR (125MHz, CDCl_3_):** δ 167.6, 161.2, 145.1, 137.2, 131.3, 129.6, 126.5, 124.5, 118.9, 114.8, 55.7, 52.7, 43.5

**Anal. Calcd for C_15_H_16_N_2_O_3_** (272.30): C, 66.16; H, 5.92; N, 10.29; **Found:** C, 65.71; H, 5.89; N, 10.42

*Methyl (E)-2-((1H-imidazol-1-yl)methyl)-3-(3,4,5-trimethoxyphenyl)acrylate* **5**

**^1^H NMR (500MHz, CDCl_3_):** δ 8.02 (s, 1H), 7.53 (s, 1H), 7.07 (s, 1H), 6.94 (s, 1H), 6.51 (s, 2H), 5.03 (s, 2H), 3.90 (s, 3H), 3.85 (s, 3H), 3.80 (s, 6H)

**^13^C NMR (125MHz, CDCl_3_):** δ 167.1, 153.5, 145.5, 139.5, 136.8, 129.5, 129.2, 125.6, 118.5, 106.3, 61.0, 56.2, 52.6, 43.4

**Anal. Calcd for C_17_H_20_N_2_O_5_** (332.36): C, 61.44; H, 6.07; N, 8.43; **Found:** C, 61.97; H, 6.08; N, 8.49

*Methyl (E)-2-((1H-imidazol-1-yl)methyl)-3-(4-nitrophenyl)acrylate* **6**

**^1^H NMR (500MHz, CDCl_3_):** δ 8.28 (d, *J* = 8.8 Hz, 2H), 8.03 (s, 1H), 7.47 (d, *J* = 8.8 Hz, 2H), 7.43 (s, 1H), 7.01 (s, 1H), 6.82 (s, 1H), 4.93 (s, 2H), 3.84 (s, 3H)

**^13^C NMR (125MHz, CDCl_3_):** δ 166.9, 143.7, 137.2, 133.0, 132.5, 130.6, 129.6, 127.9, 124.3, 118.9, 52.8, 43.1

**Anal. Calcd for C_14_H_13_N_3_O_4_** (287.28): C, 58.53; H, 4.56; N, 14.63; **Found:** C, 58.45; H, 4.54; N, 14.67

*Methyl (E)-2-((1H-imidazol-1-yl)methyl)-3-(3-nitrophenyl)acrylate* **7**

**^1^H NMR (500MHz, CDCl_3_):** δ 8.30 (d, *J* = 7.8 Hz, 1H), 8.23 (s, 1H), 8.05 (s, 1H), 7.67-7.48 (m, 3H), 7.05 (s, 1H), 6.87 (s, 1H), 4.98 (s, 2H), 3.87 (s, 3H)

**^13^C NMR (125MHz, CDCl_3_):** δ 166.2, 148.6, 141.6, 137.0, 135.5, 134.3, 130.2, 129.9, 129.7, 124.2, 123.6, 118.6, 52.9, 42.7

**Anal. Calcd for C_14_H_13_N_3_O_4_** (287.28): C, 58.53; H, 4.56; N, 14.63; **Found:** C, 58.34; H, 4.54; N, 14.68

*Methyl (E)-2-((1H-imidazol-1-yl)methyl)-3-(2-nitrophenyl)acrylate* **8**

**^1^H NMR (500MHz, CDCl_3_):** δ 8.26-8.23 (m, 2H), 7.73-7.64 (m, 2H), 7.34 (s, 1H), 7.24 (d, *J* = 7.8 Hz, 1H), 6.98 (s, 1H), 6.78 (s, 1H), 4.77 (s, 2H), 3.86 (s, 3H)

**^13^C NMR (125MHz, CDCl_3_):** δ 166.1, 147.2, 141.8, 137.1, 134.1, 130.4, 130.3, 130.1, 129.5, 128.2, 125.4, 118.7, 52.7, 43.0

*Methyl (E)-2-((1H-imidazol-1-yl)methyl)-3-(4-cyanophenyl)acrylate* **9**

**^1^H NMR (500MHz, CDCl_3_):** δ 8.02 (s, 1H), 7.76 (d, *J* = 8.3 Hz, 2H), 7.48-7.38 (m, 3H), 7.05 (s, 1H), 6.84 (s, 1H), 4.95 (s, 2H), 3.85 (s, 3H)

**^13^C NMR (125MHz, CDCl_3_):** δ 166.4, 142.5, 138.7, 137.2, 133.0, 130.0, 130.0, 129.5, 118.8, 118.2, 113.5, 53.1, 43.0

**Anal. Calcd for C_14_H_13_N_3_O_4_** (267.29): C, 67.40; H, 4.90; N, 15.72; **Found:** C, 67.34; H, 4.91; N, 15.77

*Methyl (E)-2-((1H-imidazol-1-yl)methyl)-3-(3-cyanophenyl)acrylate* **10**

**^1^H NMR (500MHz, CDCl_3_):** 7.98 (s, 1H), 7.72 (d, *J* = 7.8 Hz, 1H), 7.63-7.49 (m, 3H), 7.44 (s, 1H), 7.04 (s, 1H), 6.82 (s, 1H), 4.94 (s, 2H), δ 3.84 (s, 3H)

**^13^C NMR (125MHz, CDCl_3_):** δ 166.4, 141.9, 137.2, 135.4, 133.1, 132.8, 132.4, 130.3, 130.0, 129.9, 118.8, 118.1, 113.8, 53.0, 42.9

**Anal. Calcd for C_14_H_13_N_3_O_4_** (267.29): C, 67.40; H, 4.90; N, 15.72; **Found:** C, 67.45; H, 4.94; N, 15.68

*Methyl (E)-2-((1H-imidazol-1-yl)methyl)-3-(4-fluorophenyl)acrylate* **11**

**^1^H NMR (500MHz, CDCl_3_):** δ 8.00 (s, 1H), 7.49 (s, 1H), 7.33-7.30 (m, 2H), 7.14-7.12 (m, 2H), 7.04 (s, 1H), 6.87 (s, 1H), 4.97 (s, 2H), 3.82 (s, 3H)

**^13^C NMR (125MHz, CDCl_3_):** δ 167.0, 164.6, 162.6, 144.0, 137.2, 131.2, 130.2, 129.7, 127.1, 118.9, 116.6, 116.4, 52.8, 43.2

**Anal. Calcd for C_14_H_13_FN_2_O_2_** (260.27): C, 64.61; H, 5.03; N, 10.76; **Found:** C, 64.54; H, 5.04; N, 10.79

*Methyl (E)-2-((1H-imidazol-1-yl)methyl)-3-(4-chlorophenyl)acrylate* **12**: 65% yield;

**^1^H NMR (500MHz, CDCl_3_):** δ 7.96 (s, 1H), 7.45 (s, 1H), 7.39 (d, *J* = 8.0 Hz, 2H), 7.24 (d, *J* = 8.0 Hz, 2H), 7.01 (s, 1H), 6.84 (s, 1H), 4.94 (s, 2H), 3.80 (s, 3H)

**^13^C NMR (125MHz, CDCl_3_):** δ 166.9, 143.7, 137.2, 136.1, 132.5, 130.4, 129.7, 129.6, 127.8, 118.9, 52.8, 43.1

**Anal. Calcd for C_14_H_13_ClN_2_O_2_** (276.72): C, 60.77; H, 4.74; N, 10.12; **Found:** C, 60.47; H, 4.71; N, 9.93

*Methyl (E)-2-((1H-imidazol-1-yl)methyl)-3-(4-bromophenyl)acrylate* **13**: 70% yield;

**^1^H NMR (500MHz, CDCl_3_):** δ 7.90 (s, 1H), 7.50 (d, *J* = 8.5 Hz, 2H), 7.42 (s, 1H), 7.13 (d, *J* = 8.5 Hz, 2H), 6.97 (s, 1H), 6.80 (s, 1H), 4.89 (s, 2H), 3.75 (s, 3H)

**^13^C NMR (125MHz, CDCl_3_):** δ 166.9, 143.7, 137.2, 133.0, 132.5, 130.6, 129.6, 127.9, 124.3, 118.9, 52.8, 43.1

**Anal. Calcd for C_14_H_13_BrN_2_O_2_** (321.17): C, 52.36; H, 4.08; N, 8.72; **Found:** C, 52.54; H, 4.07; N, 8.75

*Methyl (E)-2-((1H-1,2,4-triazol-1-yl)methyl)-3-phenylacrylate* **14**

**^1^H NMR (500MHz, CDCl_3_):** δ 8.25 (s, 1H), 8.08 (s, 1H), 7.98 (s, 1H), 7.71 (d, *J* = 6.8 Hz, 2H), 7.51-7.38 (m, 3H), 5.21 (s, 2H), 3.83 (s, 3H)

**^13^C NMR (125MHz, CDCl_3_):** δ 167.2, 152.0, 145.8, 144.3, 133.9, 130.6, 129.5, 129.0, 125.4, 52.7, 46.1

**Anal. Calcd for C_13_H_13_N_3_O_2_** (243.27): C, 64.19; H, 5.39; N, 17.27; **Found:** C, 64.53; H, 5.41; N, 17.32

*Methyl (E)-2-((1H-1,2,4-triazol-1-yl)methyl)-3-(naphthalen-1-yl)acrylate* **15**

**^1^H NMR (500MHz, CDCl_3_):** δ 8.62 (s, 1H), 8.19 (s, 1H), 8.01-7.90 (m, 5H), 7.61-7.53 (m, 3H), 5.15 (s, 2H), 3.89 (s, 3H)

**^13^C NMR (125MHz, CDCl_3_):** δ 166.8, 137.8, 144.1, 133.7, 131.6, 131.1, 130.3, 129.0, 127.7, 127.4, 127.2, 126.8, 125.7, 124.4, 52.8, 46.7

**Anal. Calcd for C_17_H_15_N_3_O_2_** (293.33): C, 69.61; H, 5.15; N, 14.33; **Found:** C, 69.77; H, 5.27; N, 14.56

*Methyl (E)-2-((1H-1,2,4-triazol-1-yl)methyl)-3-(p-tolyl)acrylate* **16**

**^1^H NMR (500MHz, CDCl_3_):** δ 8.25 (s, 1H), 8.06 (s, 1H), 7.99 (s, 1H), 7.61 (d, *J* = 8.0, 2H), 7.28 (d, *J* = 8.5, 2H), 5.23 (s, 2H), 3.83 (s, 3H), 2.41 (s, 3H)

**^13^C NMR (125MHz, CDCl_3_):** δ 167.4, 152.0, 146.0, 144.2, 140.7, 131.1, 130.0, 129.9, 124.3, 52.7, 46.5, 21.7

**Anal. Calcd for C_14_H_15_N_3_O_2_** (257.29): C, 65.36; H, 5.88; N, 16.33; **Found:** C, 65.51; H, 5.86; N, 16.39

*Methyl (E)-2-((1H-1,2,4-triazol-1-yl)methyl)-3-(4-methoxyphenyl)acrylate* **17**

**^1^H NMR (500MHz, CDCl_3_):** δ 8.27 (s, 1H), 8.04 (s, 1H), 8.00 (s, 1H), 7.72 (d, *J* = 6.8 Hz, 2H), 7.00 (d, *J* = 6.8 Hz, 2H), 5.25 (s, 2H), 3.87 (s, 3H), 3.83 (s, 3H)

**^13^C NMR (125MHz, CDCl_3_):** δ 167.3, 161.2, 151.8, 145.4, 144.0, 131.8, 126.2, 122.6, 114.4, 55.4, 52.4, 46.4

**Anal. Calcd for C_14_H_15_N_3_O_3_** (273.29): C, 61.53; H, 5.53; N, 15.38; **Found:** C, 61.76; H, 5.51; N, 15.34

*Methyl (E)-2-((1H-1,2,4-triazol-1-yl)methyl)-3-(3,4,5-trimethoxyphenyl)acrylate* **18**: 88% yield; **^1^H NMR (500MHz, CDCl_3_):** δ 8.32 (s, 1H), 8.00 (s, 1H), 7.95 (s, 1H), 7.14 (s, 2H), 5.24 (s, 2H), 3.91 (s, 9H), 3.84 (s, 3H)

**^13^C NMR (125MHz, CDCl_3_):** δ 167.1, 153.4, 151.6, 145.8, 144.4, 139.5, 129.0, 124.2, 107.3, 60.9, 56.4, 52.5, 46.3

**Anal. Calcd for C_16_H_19_N_3_O_5_** (333.34): C, 57.65; H, 5.75; N, 12.61; **Found:** C, 57.84; H, 5.73; N, 12.52

*Methyl (E)-2-((1H-1,2,4-triazol-1-yl)methyl)-3-(4-nitrophenyl)acrylate* **19**: 80% yield;

**^1^H NMR (500MHz, CDCl_3_):** δ 8.34 (d, *J* = 6.3 Hz, 2H), 8.30 (s, 1H), 8.07 (s, 1H), 8.02 (d, *J* = 8.8 Hz, 2H), 7.99 (s, 1H), 5.14 (s, 2H), 3.85 (s, 3H)

**^13^C NMR (125MHz, CDCl_3_):** δ 166.3, 152.2, 148.5, 144.8, 142.7, 140.3, 130.7, 128.6, 124.3, 53.1, 46.0

*Methyl (E)-2-((1H-1,2,4-triazol-1-yl)methyl)-3-(3-nitrophenyl)acrylate* **20**: 75% yield;

**^1^H NMR (500MHz, CDCl_3_):** δ 8.66 (s, 1H), 8.32-8.25 (m, 3H), 8.07 (s, 1H), 8.00 (s, 1H), 7.70 (t, *J* = 8.1 Hz, 1H), 5.16 (s, 2H), 3.86 (s, 3H)

**^13^C NMR (125MHz, CDCl_3_):** δ 166.2, 152.0, 148.6, 144.5, 142.3, 135.3, 135.3, 130.1, 127.9, 124.5, 124.3, 52.8, 45.6

**Anal. Calcd for C_13_H_12_N_3_O_4_** (288.26): C, 54.17; H, 4.20; N, 19.44; **Found:** C, 54.24; H, 4.19; N, 19.95

*Methyl (E)-2-((1H-1,2,4-triazol-1-yl)methyl)-3-(2-nitrophenyl)acrylate* **21**: 76% yield;

**^1^H NMR (500MHz, CDCl_3_):** δ 8.21 - 8.31 (m, 3H), 8.09 (d, *J* = 7.3 Hz, 1H), 7.93 (s, 1H), 7.80 (t, *J* = 7.6 Hz, 1H), 7.62 (t, *J* = 8.0 Hz, 1H), 4.99 (s, 2H), 3.84 (s, 3H)

**^13^C NMR (125MHz, CDCl_3_):** δ 166.0, 151.9, 147.5, 144.4, 142.1, 134.2, 131.6, 130.2, 130.0, 126.6, 125.2, 52.7, 46.0

**Anal. Calcd for C_13_H_12_N_3_O_4_** (288.26): C, 54.17; H, 4.20; N, 19.44; **Found:** C, 54.12; H, 4.22; N, 19.86

*Methyl (E)-2-((1H-1,2,4-triazol-1-yl)methyl)-3-(4-cyanophenyl)acrylate* **22**: 57% yield;

**^1^H NMR (500MHz, CDCl_3_):** δ 8.30 (s, 1H), 8.03 (s, 1H), 7.99 (s, 1H), 7.95 (d, *J* = 8.8 Hz, 2H), 7.79 (d, *J* = 8.3 Hz, 2H), 5.14 (s, 2H), 3.85 (s, 3H)

**^13^C NMR (125MHz, CDCl_3_):** δ 166.4, 152.2, 144.8, 143.1, 138.4, 132.8, 130.4, 128.1, 118.5, 113.5, 53.0, 46.0

*Methyl (E)-2-((1H-1,2,4-triazol-1-yl)methyl)-3-(3-cyanophenyl)acrylate* **23**: 58% yield;

**^1^H NMR (500MHz, CDCl_3_):** δ 8.30 (s, 1H), 8.15-8.11 (m, 2H), 8.00 (s, 2H), 7.74 (d, *J* = 8.0 Hz, 1H), 7.63 (t, *J* = 8.0 Hz, 1H), 5.13 (s, 2H), 3.85 (s, 3H)

**^13^C NMR (125MHz, CDCl_3_):** δ 166.4, 152.2, 144.8, 142.7, 135.3, 133.9, 133.2, 133.2, 130.1, 127.9, 118.4, 113.6, 53.0, 45.9

**Anal. Calcd for C_13_H_12_N_4_O_2_** (268.28): C, 62.68; H, 4.51; N, 20.88; **Found:** C, 62.39; H, 4.54; N, 20.95

*Methyl (E)-2-((1H-1,2,4-triazol-1-yl)methyl)-3-(4-fluorophenyl)acrylate* **24**: 55% yield;

**^1^H NMR (500MHz, CDCl_3_):** δ 8.28 (s, 1H), 8.03 (s, 1H), 7.98 (s, 1H), 7.80-7.78 (m, 2H), 7.17 (t, *J* = 8.5 Hz, 2H), 5.19 (s, 2H), 3.83 (s, 3H)

**^13^C NMR (125MHz, CDCl_3_):** δ 167.1, 164.8, 162.8, 152.1, 144.5, 132.1, 130.1, 125.2, 116.4, 116.3, 52.8, 46.2

**Anal. Calcd for C_13_H_12_FN_3_O_2_** (261.26): C, 59.77; H, 4.63; N, 16.08; **Found:** C, 59.96; H, 4.69; N, 16.00

*Methyl (E)-2-((1H-1,2,4-triazol-1-yl)methyl)-3-(4-chlorophenyl)acrylate* **25**: 78% yield;

**^1^H NMR (500MHz, CDCl_3_):** δ 8.24 (s, 1H), 7.91 (d, *J* = 7.8 Hz, 2H), 7.67 (d, *J* = 7.8 Hz, 2H), 7.38 (d, *J* = 7.8 Hz, 2H), 5.11 (s, 2H), 3.76 (s, 3H)

**^13^C NMR (125MHz, CDCl_3_):** δ 166.8, 151.9, 144.5, 144.2, 136.2, 132.3, 131.2, 129.4, 125.9, 52.7, 46.1

**Anal. Calcd for C_13_H_12_ClN_3_O_2_** (277.71): C, 56.23; H, 4.36; N, 15.13; **Found:** C, 56.32; H, 4.29; N, 15.20

*Methyl (E)-2-((1H-1,2,4-triazol-1-yl)methyl)-3-(4-bromophenyl)acrylate* **26**

**^1^H NMR (500MHz, CDCl_3_):** δ 8.28 (s, 1H), 7.99 (d, *J* = 3.4 Hz, 2H), 7.69-7.60 (m, 4H), 5.17 (s, 2H), 3.84 (s, 3H)

**^13^C NMR (125MHz, CDCl_3_):** δ 166.9, 152.1, 144.5, 132.8, 132.4, 131.4, 130.0, 126.0, 124.7, 52.8, 46.2
